# Establishment and validation of a novel anoikis-related prognostic signature of clear cell renal cell carcinoma

**DOI:** 10.3389/fimmu.2023.1171883

**Published:** 2023-03-28

**Authors:** Yankuo Liu, Zhiyuan Shi, Jianzhong Zheng, Zeyuan Zheng, Huimin Sun, Zuodong Xuan, Yang Bai, Meiling Fu, Yifan Du, Chen Shao

**Affiliations:** ^1^Department of Urology, Xiang’an Hospital of Xiamen University, School of Medicine, Xiamen University, Xiamen, China; ^2^Central Laboratory, Xiang’an Hospital of Xiamen University, School of Medicine, Xiamen University, Xiamen, China

**Keywords:** anoikis, clear cell renal cell carcinoma, prognostic model, tumor microenvironment, bioinformatics

## Abstract

**Background:**

Despite progression in its treatment, the clinical outcome of patients with clear cell renal cell carcinoma (ccRCC) remains not ideal. Anoikis is a unique form of programmed apoptosis, owing to insufficient cell-matrix interactions. Anoikis plays a crucial role in tumor migration and invasion, and tumor cells could protect themselves through the capacity of anoikis resistance.

**Methods:**

Anoikis-related genes (ARGs) were obtained from Genecards and Harmonizome portals. The ARGs related to ccRCC prognosis were identified through univariate Cox regression analysis, then we utilized these ARGs to construct a novel prognostic model for ccRCC patients. Moreover, we explored the expression profile of ARGs in ccRCC using the Cancer Genome Atlas (TCGA) and Genotype-Tissue Expression (GTEx) database. We also conducted Real-Time Polymerase Chain Reaction (RT-PCR) to probe ARGs expression of the risk score. Finally, we performed correlation analysis between ARGs and tumor immune microenvironment.

**Results:**

We identified 17 ARGs associated with ccRCC survival, from which 7 genes were chosen to construct a prognostic model. The prognostic model was verified as an independent prognostic indicator. The expression of most ARGs was higher in ccRCC samples. These ARGs were closely correlated with immune cell infiltration and immune checkpoint members, and had independent prognostic value respectively. Functional enrichment analysis demonstrated that these ARGs were significantly associated with multiple types of malignances.

**Conclusion:**

The prognostic signature was identified to be highly efficient in predicting ccRCC prognosis, and these ARGs were closely related to tumor microenvironment.

## Introduction

Renal cell carcinoma (RCC) is one of the predominant tumors in urological system, and there is an increasing number of new patients with RCC year by year (1, 2). Clear cell RCC (ccRCC) is the most frequent pathological subtype of RCC, with a ratio of 75-80% (3). The clinical prognosis of RCC patients is closely associated with disease pathological stage, and relapse-free survival rate is decreasing from stage I to stage IV (4). Early surgical resection is considered as the predominant treatment for ccRCC patients (5). However, about 40% of patients with surgical treatment occur distant metastases, contributing to poor endpoint (4). For patients with metastatic or unresectable RCC, targeted therapy drugs were demonstrated to prolong survival time effectively, including sorafenib and sunitinib (6, 7). However, various of adverse effects decrease the life quality of patients during target treatment, such as hypertension (8). Thus, there is an urgent need to perform early diagnosis of ccRCC, finding novel diagnosis biomarkers and prognostic signatures have great clinical benefit.

Epithelial cells in human body keep normal tissue structure by cell-to-cell adherence and cell-to-extracellular matrix interactions (9). Four cell adhesion protein molecules are involved in these interactions, including: cadherins, immunoglobulins, selectins, and integrins. Anoikis is a kind of specific programmed apoptosis which occurs when cell-to-cell adherence and cell-to-extracellular matrix interactions are destroyed, and it plays an important role in maintaining tissue homeostasis by killing misplaced cells (10). Mitochondria- and cell surface death receptor-mediated pathways are necessary for triggering anoikis (11). In diverse tumors, anoikis serves as a protective physiological barrier inhibiting dissemination of metastatic cancer cells. However, cancer cells have the power of anoikis resistance to guarantee their survival *via* different signaling pathways, resulting in the distant metastasis (12). Previous studies have demonstrated that anoikis-related genes (ARGs) were closely correlated with metastatic cascade and progression of cancer. As a member of ARGs, the expression of Fas apoptotic inhibitory molecule 2 (FAIM2) was positively linked to tumor stage and poor endpoint of non-small cell lung cancer, and FAIM2 knockdown may inhibit anoikis resistance (13). Kruppel-like factor 5 (KLF5) was verified to promote anoikis resistance, and elevated KLF5 expression was correlated with poor clinical outcome in colorectal cancer patients (14).

Past studies have validated that several ARGs were involved in reversing anoikis resistance and invasion and migration in RCC (15, 16). Nevertheless, few studies have systematically explored the role of ARGs in ccRCC. In our study, multiple databases are integrated to screen seven ARGs. Then, the anoikis prognostic model (APM) based on these seven ARGs was constructed and validated. Finally, we performed several analyses to explore the expression profile of these ARGs and the correlation between immune cell infiltration and ARGs expression in ccRCC.

## Materials and methods

### Data collection and processing

We downloaded the GSE53757 (72 normal renal samples and 72 ccRCC samples), GSE40435 (101 normal renal samples and 101 ccRCC samples), and GSE66272 (27 normal renal samples and 27 ccRCC samples) datasets from the GEO database (https://www.ncbi.nlm.nih.gov/geo/, accessed on 12 October 2022). Then we obtained 316 anoikis-related genes from the genecards website (17) (https://www.genecards.org/, accessed on 12 October 2022) and Harmonizome portals (18) (https://maayanlab.cloud/Harmonizome/, accessed on 12 October 2022). The above data analysis was performed by R software. The ARGs expression data was normalized *via* the “edgeR” package (19). We obtained the differentially expressed genes (DEGs) between ccRCC tissues and normal kidney samples in the GSE53757, GSE40435, GSE66272, and KIRC datasets by “limma” package. Furthermore, we collected the tumor prognostic genes (TPGs) *via* “survival” package, and overall survival (OS) of these genes was verified using univariate Cox analysis (20).

### Construction and validation of the APM

We utilized the least absolute shrinkage and selection operator (LASSO) Cox regression analysis to screen out ARGs which were closely associated with prognosis of ccRCC patients, and optimal lambda (λ) was seen as the optimal value *via* cross validation (21, 22). Then we computed the risk score by integrating the expression profile of ARGs and paired multivariate Cox regression values (β). We divided the ccRCC patients into two groups (low-risk group and high-risk group) based on the media risk score. The “umap” package and “Rtsne” package were used to demonstrate the distribution patterns of ccRCC patients. We utilized the “survminer” package to perform Kaplan-Meier (K-M) survival analysis. The “time” ROC package was used to perform time-dependent receiver operating characteristic (ROC) analysis.

### Predictive role of the APM

A heatmap was employed to demonstrate the APM and clinical characteristics including age, grade, gender, and stage of patients. We used the univariate and multivariate Cox regression analyses to evaluate the predictive value of the APM and several clinical factors.

### Construction and validation of the nomogram

The “survival” package and “rms” package were employed to build a nomogram predicting prognosis based on the Cox regression analysis results. We used the calibration curve to estimate the predictive effectiveness of the nomogram. The “time” ROC package was utilized to perform the time-dependent ROC analysis which could estimate the precision of the APM.

### Gene expression analysis of ARGs

In our study, we utilized R (version3.6.3) to analyze the mRNA expression levels of the ARGs in tumor tissues and paracancerous tissue samples in The Cancer Genome Atlas (TCGA) and normal kidney tissues in Genotype-Tissue Expression (GTEx) database which were downloaded from UCSC Xena website (https://xena.ucsc.edu/) (23). The expression profile was visualized with ggplot2 (3.3.3). Then we evaluated the statistical significance of the differential expression by using the Wilcoxon test. The total protein expression levels of ARGs were collected from Clinical Proteomic Tumor Analysis Consortium (CPTAC) dataset in the UALCAN website (http://ualcan.path.uab.edu/analysis-prot.html), a convenient tool for exploring cancer omics data (24).

### Immune infiltration analysis

R package “estimate” was employed to investigate the abundance of stromal components (StromalScore), immune cell infiltration levels (ImmuneScore) and ESTIMATEScore of ccRCC. The associations between ARGs expression and immune/stromal/estimate scores were evaluated by Spearman’s correlation analysis. The CIBERSORT algorithm was utilized to conduct the immune cell infiltration estimations (25). The partial correlation (cor) values and *p* values were calculated *via* the purity-adjusted Spearman’s rank correlation test.

### Functional and pathway enrichment analysis

With the help of GeneMANIA website (http://www.genemania.org), we explored and displayed the interaction network among these seven ARGs. We used WebGestalt to perform Gene Ontology (GO) and Kyoto Encyclopedia of Genes and Genomes (KEGG) analysis about ARGs in ccRCC.

### Cell culture

We obtained three human ccRCC cell lines 786-O, Caki-1 and ACHN and human renal proximal convoluted tubule cell HK2 from American Type Cultural Collection (ATCC). 786-O and Caki-1 were cultured in RPMI 1640 media (11875119, Gibco, Foster, CA, USA). HK2 and ACHN were kept in DMEM high glucose media (11965092, Gibco, Foster, CA, USA). All media contained 1% penicillin-streptomycin (15140163, Gibco, Foster, CA, USA) solution and 10% fetal bovine serum (FBS) (10100147, Gibco, Foster, CA, USA). All cells were maintained in an incubator at 37°C with a 5% CO_2_.

### Reverse transcription and PCR analysis

Total RNA from cells was extracted by using the Spark easy cellular RNA extraction kit (AC0205) (Shandong Sparkjade Biotechnology Co., Ltd). The Evo M-MLV RT Kit (AG11711) was obtained from Accurate Biotechnology (Hunan) Co., Ltd. The Hieff^®^qPCR SYBR Green Master Mix (11201ES08) were obtained from Yeasen Biotechnology (Shanghai) Co., Ltd. (Shanghai, China). The primer sequences of RT-qPCR were displayed in **Table S1**.

### Statistical analysis

All data were demonstrated by mean ± standard deviation. Statistical analysis was conducted by using SPSS 13. The value of *p* < 0.05 was considered significant (* *p* < 0.05, ** *p* < 0.01, *** *p* < 0.001, **** *p* < 0.0001). The “ns” represented no statistically significant.

## Results

### Identification of the ARGs

**Figure 1:**
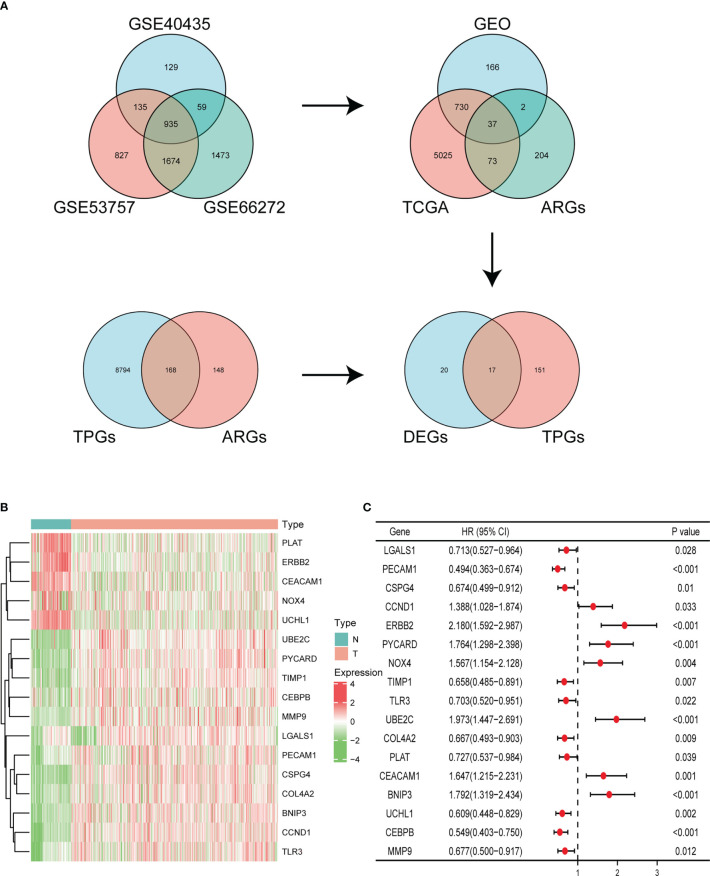
Identification of ARGs. **(A)** Identifying differentially expressed ARGs associated with OS *via* Venn diagram. **(B)** Heatmap of the expression of 17 ARGs in KIRC dataset of TCGA and GTEx. **(C)** Forest plot displaying univariate Cox regression analysis of 17 ARGs in ccRCC patients.

In our study, a total of 935 DEGs were identified from three GEO databases, including GSE40435, GSE53757, and GSE66272. Then we obtained 37 significant DEGs from GEO dataset, TCGA database, and ARGs. In addition, we identified 168 TPGs based on ARGs and TPGs database. Finally, we integrated the 37 significant DEGs and 168 TPGs and screened out 17 genes (**Figure 1A**). In these genes, 12 genes expression were upregulated in ccRCC tissues compared with normal kidney samples in TCGA and GTEx, including UBE2C, PYCARD, TIMP1, CEBPB, MMP9, LGALS1, PECAM1, CSPG4, COL4A2, BNIP3, CCND1, TLR3. While 5 genes were downregulated in tumor tissues, including PLAT, ERBB2, CEACAM1, NOX4, and UCHL1 (**Figure 1B**). Univariate Cox regression analysis demonstrated that 17 ARGs were closely linked to clinical outcome of ccRCC patients (**Figure 1C**).

### Construction of the APM

**Figure 2:**
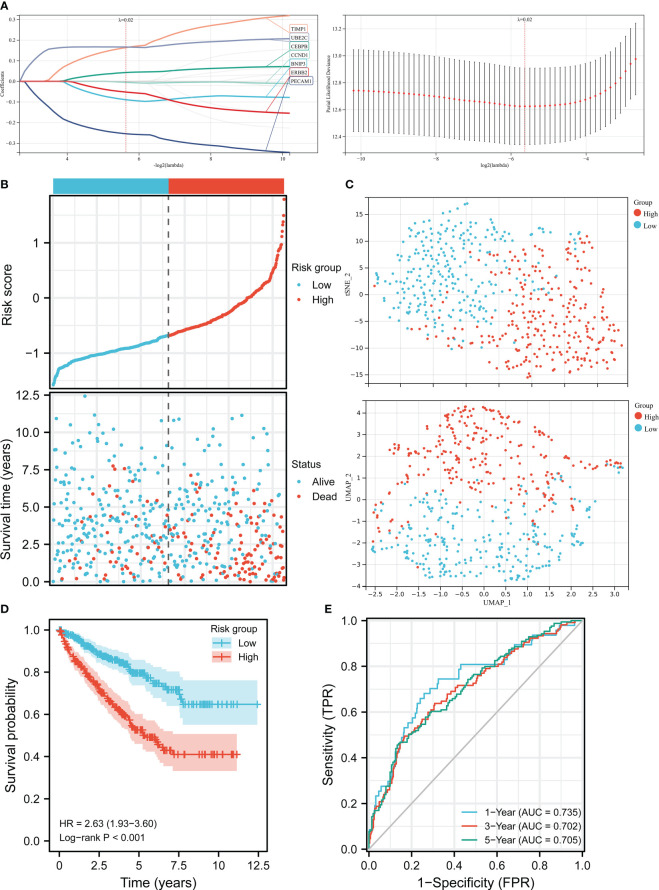
Construction of the risk score model in TCGA. **(A)** LASSO regression analysis and LASSO coefficient profiles of ARGs. **(B)** Risk score distribution and ccRCC patients’ survival status. **(C)** UMAP plot and t-SNE plot of the ccRCC patients exhibiting the distribution in high and low-risk groups. **(D)** K-M curves of patients in high and low-risk groups. **(E)** AUC of the time-dependent ROC curves.

LASSO regression analysis was employed to identify seven prognosis-related genes including BNIP3, CCND1, CEBPB, ERBB2, PECAM1, TIMP1, and UBE2C from 17 ARGs (**Figure 2A**). By weighting the expression of these seven genes with LASSO regression coefficient, we constructed the APM based on the following algorithm. Risk score = (-0.08912 * BNIP3 expression) + (-0.00457 * CCND1 expression) + (0.04290 * CEBPB expression) + (-0.05204 * ERBB2 expression) + (-0.25383 * PECAM1 expression) + (0.16386 * TIMP1 expression) + (0.16675 * UBE2C expression). We firstly checked the clinical information and gene expression profiles of the ccRCC patients in TCGA, 13 cases without gene expression and unabridged clinical data were deleted. Then 526 patients were grouped into a high-risk cluster (n = 263) and a low-risk cluster (n = 263) according to the media risk score. The survival time of patients in high-risk group was shorter than patients in low-risk group (**Figure 2B**). The UMAP and t-SNE analysis demonstrated that the distribution of ccRCC patients in two groups was in line with the expectation (**Figure 2C**). The K-M curve indicated that the OS of high-risk group was lower than that of low-risk group (**Figure 2D**). ROC analysis was employed to evaluate the predictive performance of the APM, and the area under curve (AUC) values in 1-year, 3-year, 5-year were 0.735, 0.702, and 0.705, respectively, suggesting that the APM could be an ideal prognosis indicator (**Figure 2E**).

### Independent prognostic value of the APM and construction of the nomogram

**Figure 3:**
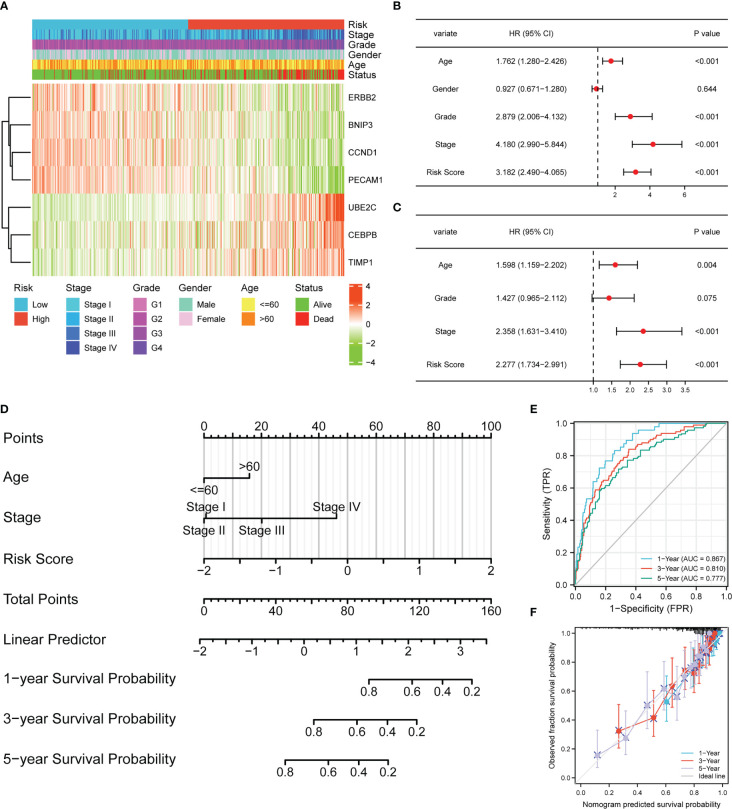
The risk score model could be an independent prognostic indicator for ccRCC. **(A)** Heatmap demonstrating the association between ARGs expression and clinical factors and risk groups. **(B)** Univariate Cox regression analysis of ccRCC patients in TCGA. **(C)** Multivariate Cox regression analysis of ccRCC patients in TCGA. **(D)** Nomogram-based gene signature for predicting 1-, 3- and 5-year OS. ROC curves **(E)** and calibration curves **(F)** for predicting the 1-, 3-, and 5-year performance of the nomogram.

The heatmap demonstrated the correlation between risk group, stage, grade, gender, age, status, and expression levels of seven ARGs (**Figure 3A**). A higher risk score was found to be associated with higher expression levels of UBE2C, CEBPB, TIMP1, and lower expression levels of ERBB2, BNIP3, CCND1, PECAM1. A univariate Cox regression analysis indicated that age, cancer grade, risk score and tumor stage were closely correlated with OS (**Figure 3B**). Meanwhile, a multivariate Cox regression analysis displayed that risk score, age, and tumor stage were significantly related to OS (**Figure 3C**). These results indicated that the APM had independent prognostic value in ccRCC patients. Next, we constructed a nomogram about age, stage, and risk score to compute the survival probability rates (**Figure 3D**). The 1-, 3-, and 5-year AUCs for the nomogram were 0.867, 0.810, and 0.777, respectively (**Figure 3E**). Furthermore, the calibration plot also demonstrated the ideal prediction effect at 1-, 3-, and 5-year OS (**Figure 3F**).

### Validation of the APM in E-MTAB-1980 cohort

**Figure 4:**
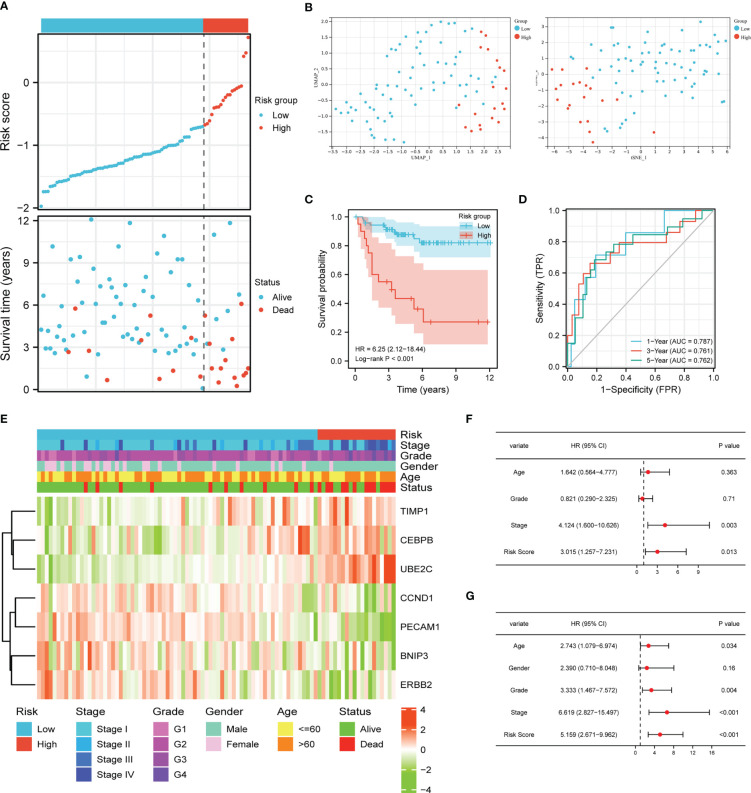
Validation of the risk score model. **(A)** Risk score distribution and ccRCC patients’ survival status. **(B)** UMAP plot and t-SNE plot of the ccRCC patients exhibiting the distribution in high and low-risk groups. **(C)** K-M curves of patients in high and low-risk groups. **(D)** AUC of the time-dependent ROC curves. **(E)** Heatmap demonstrating the association between ARGs expression and clinical factors and risk groups. Univariate Cox regression analysis **(F)** and multivariate Cox regression analysis **(G)** of ccRCC patients.

The clinical feature and gene expression profile of the patients in E-MATB-1980 cohort were checked, and patients with abridged information were deleted from the cohort. Then we chose 92 cases to verify the prognostic value of the APM, which were divided into a low-risk group (n = 33) and a high-risk group (n = 59) based on the media risk score. **Figure 4A** demonstrated that most dead patients were distributed into the high-risk group, suggesting the ideal predictive value of the risk score. UMAP and t-SNE analysis displayed that the ccRCC patients of two groups could be placed into two sets suitably (**Figure 4B**). The K-M curve indicated that the high-risk group patients had a worse clinical outcome, and the AUC values in 1-year, 3-year, 5-year were 0.787, 0.761, and 0.762, respectively (**Figures 4C, D**). The heatmap demonstrated the correlation between expression levels of these seven ARGs and risk group, clinical information, and pathological characteristic (**Figure 4E**). The univariate Cox regression analysis showed that stage and risk score were closely related to OS (**Figure 4F**). Meanwhile, the age, grade, stage, and risk score had an intimate relationship with OS in multivariate Cox regression (**Figure 4G**). These results further verified the independent prognostic value of the APM.

### Expression profiles of ARGs in ccRCC

**Figure 5:**
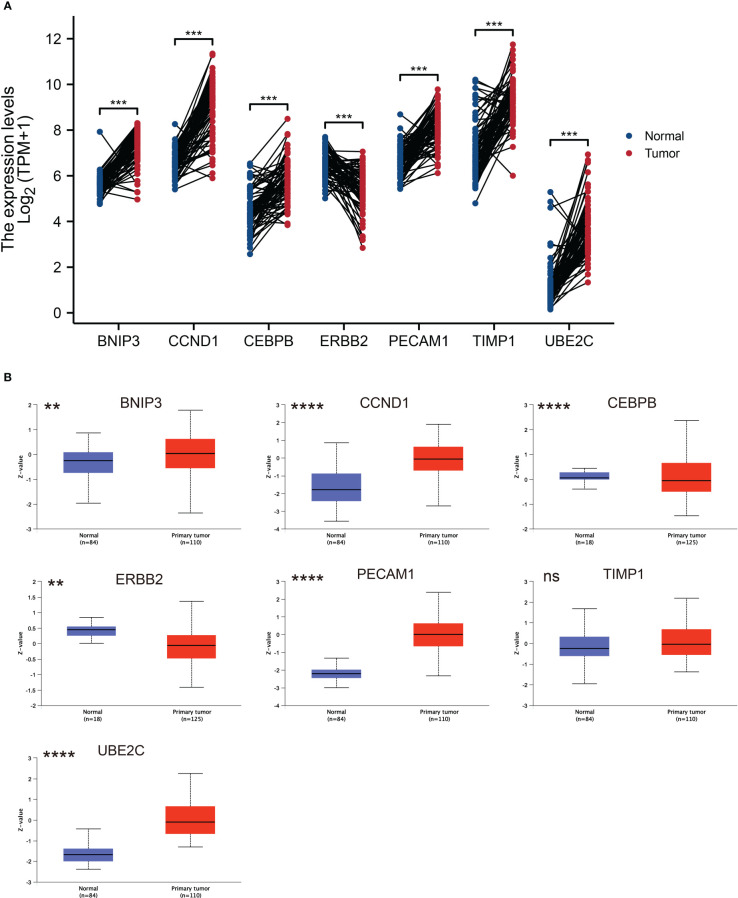
ARGs expression profiles in ccRCC and normal tissues. **(A)** Expression levels of ARGs in ccRCC and paired normal tissue samples. The red color represents tumor tissue, while the blue color represents normal tissue. **(B)** Total protein levels of ARGs in ccRCC and normal tissue samples from CPTAC. ** p < 0.01, *** p < 0.001, **** p < 0.0001. ns represents no significance.

Next, we explored the expression patterns of these seven ARGs in ccRCC tissues and paired normal kidney samples based on TCGA dataset. As graphed in **Figure 5A**, the mRNA expression levels of BNIP3, CCND1, CEBPB, PECAM1, TIMP1, and UBE2C were higher than that in normal kidney tissues, while ERBB2 was downregulated in ccRCC. The protein expression levels of BNIP3, CCND1, PECAM1, TIMP1, and UBE2C were elevated in ccRCC, while CEBPB and ERBB2 protein expression were downregulated in ccRCC (**Figure 5B**).

**Figure 6:**
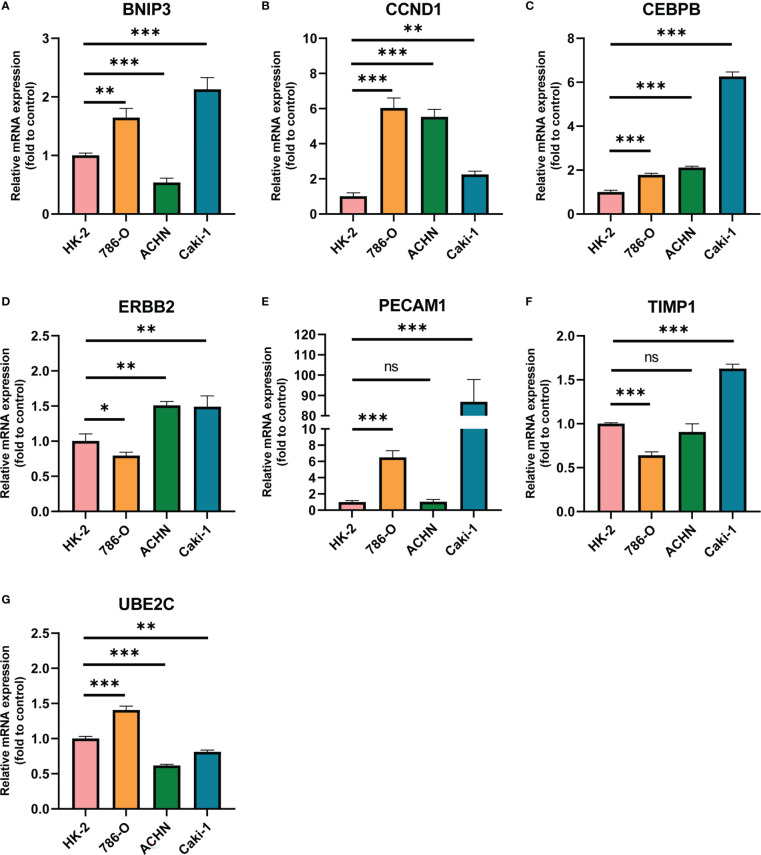
The mRNA expression of ARGs in HK2 and three ccRCC cell lines quantified by real-time PCR. **(A)** BNIP3; **(B)** CCND1; **(C)** CEBPB; **(D)** ERBB2; **(E)** PECAM1; **(F)** TIMP1; **(G)** UBE2C. * p < 0.05, ** p < 0.01, *** p < 0.001, **** p < 0.0001. ns represents no significance.

Furthermore, we chose human ccRCC cell lines 786-O, Caki-1 and ACHN and human renal proximal convoluted tubule cell HK2 to detect the expression of ARGs *via* RT-qPCR experiments. As displayed in **Figure 6**, the mRNA expression levels of CCND1 and CEBPB were higher in three ccRCC cell lines than that in HK2, and BNIP3 and PECAM1 were higher expressed in 786-O and Caki-1 than HK2. We also conducted immunohistochemistry (IHC) staining to investigate the UBE2C protein level in ccRCC and par-carcinoma tissue. The result demonstrated that UBE2C protein level was significantly upregulated in ccRCC compared to par-carcinoma tissue, which was consistent with previous result from CPTAC (**Figure S1**).

### Clinicopathological stages and independent prognostic value of ARGs in ccRCC

As displayed in **Figure S2**, the expression levels of these seven ARGs were strongly correlated with pathological stages of ccRCC patients. Notably, the expression levels BNIP3, CCND1, ERBB2, and PECAM1 were downregulated from stage I to stage IV in ccRCC, while expression of CEBPB, TIMP1, and UBE2C were elevated in higher stage. To further explore the independent prognostic value of single ARGs in ccRCC, we performed survival analysis using K-M plotter. The result demonstrated that high expression levels of BNIP3, CCND1, ERBB2, and PECAM1 were correlated with better clinical endpoint of ccRCC, while high CEBPB, TIMP1, and UBE2C expression were linked to worse clinical outcome (**Figure S3**). The K-M result was consistent with previous Cox regression analysis.

### TME and immune infiltration analysis of ARGs

**Figure 7:**
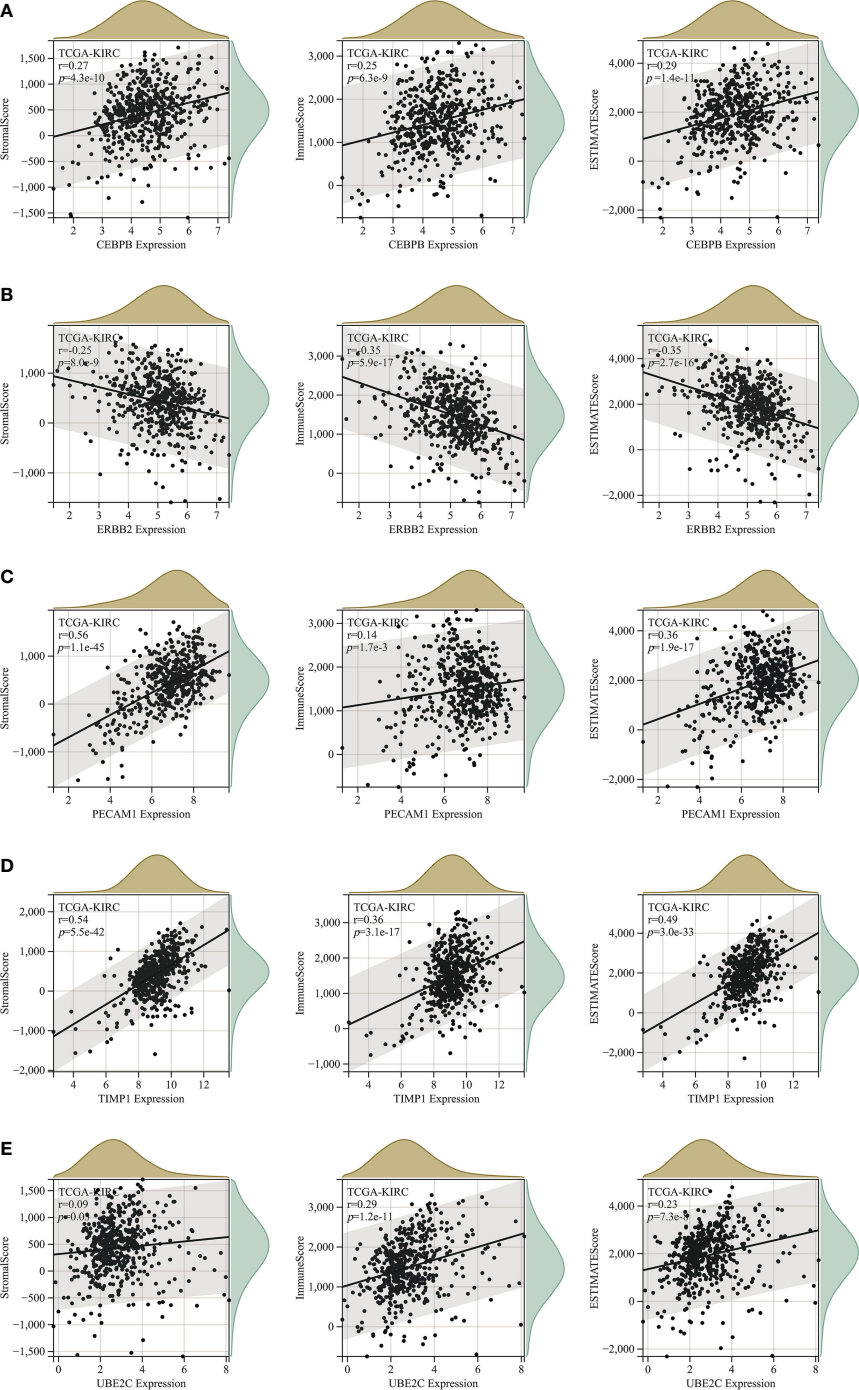
The correlation analysis between StromalScore, ImmuneScore and ESTIMATEScore and expression levels of **(A)** CEBPB; **(B)** ERBB2; **(C)** PECAM1; **(D)** TIMP1; **(E)** UBE2C.

**Figure 8:**
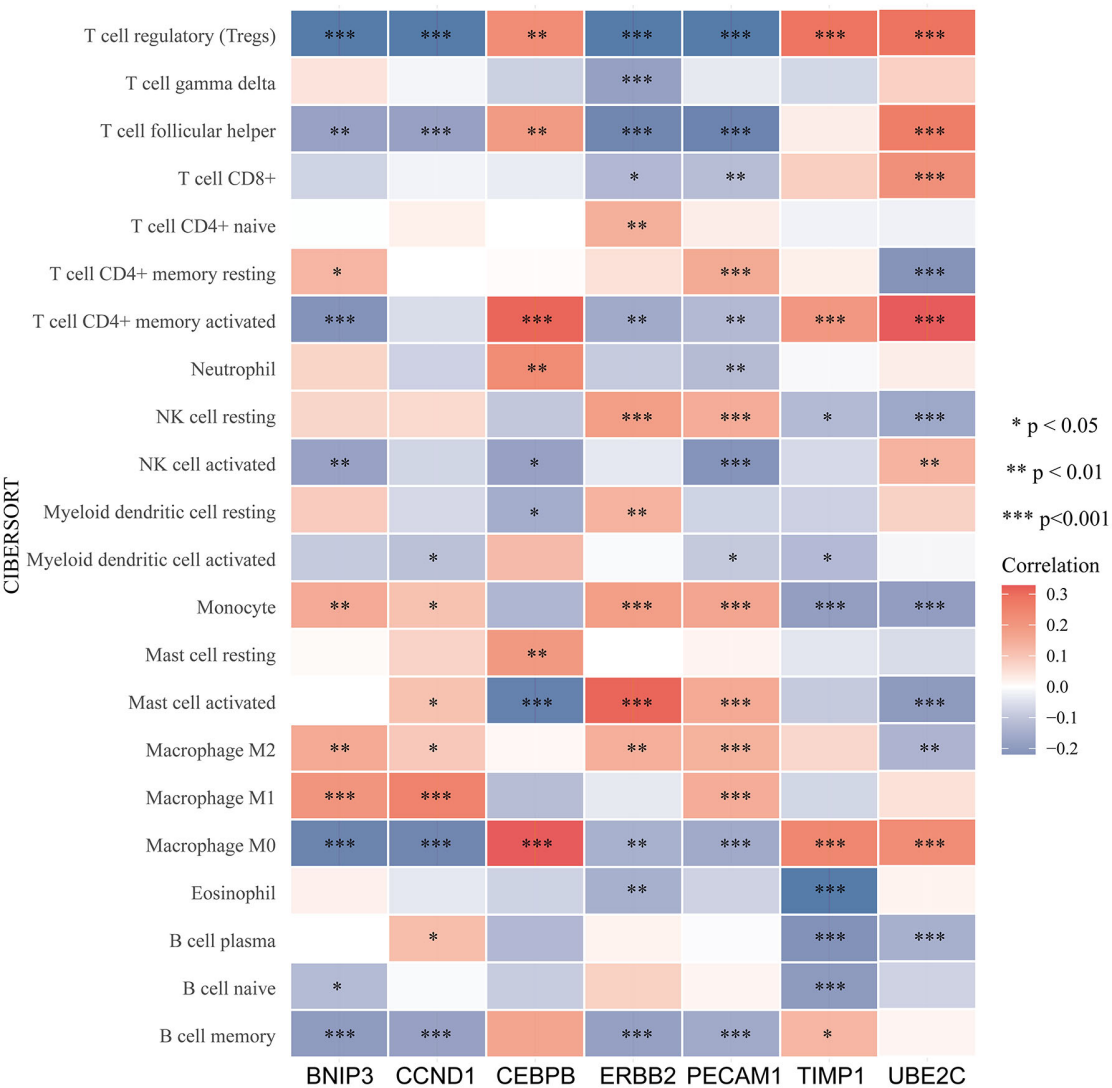
Relationship between immune cells infiltration and ARGs expression based on CIBERSORT. The red color indicates a positive correlation, while the blue color represents a negative correlation.

An increasing number of evidences have illustrated that tumor microenvironment (TME) plays a significant role in tumor occurrence and progression (26, 27). Therefore, it is essential to dig out the relationship between expression of ARGs and TME. In our study, the ESTIMATE algorithm was utilized to evaluate the immune and stromal scores of ccRCC. **Figure 7** demonstrated that the expression of CEBPB, PECAM1, TIMP1, and UBE2C were significantly positively associated with stromal score, as well as with immune score, while ERBB2 expression was negatively correlated with these scores. The relationship between BNIP3 and CCND1 had no statistical significance (**Figure S4**). Moreover, we examined the association between expression of ARGs and immune cell infiltration level in ccRCC using CIBERSORT algorithm. As displayed in **Figure 7**, the expression of BNIP3, CCND1, ERBB2, and PECAM1 were closely negatively related to infiltration levels of regulatory T cell, follicular helper T cell, M0 macrophage, and memory B cell, and were positively correlated with M2 macrophage and monocyte. CEBPB, TIMP1, and UBE2C expression levels were strongly positively associated with infiltration levels of regulatory T cell, CD4+ memory activated T cell, and M0 macrophage (**Figure 8**).

**Figure 9:**
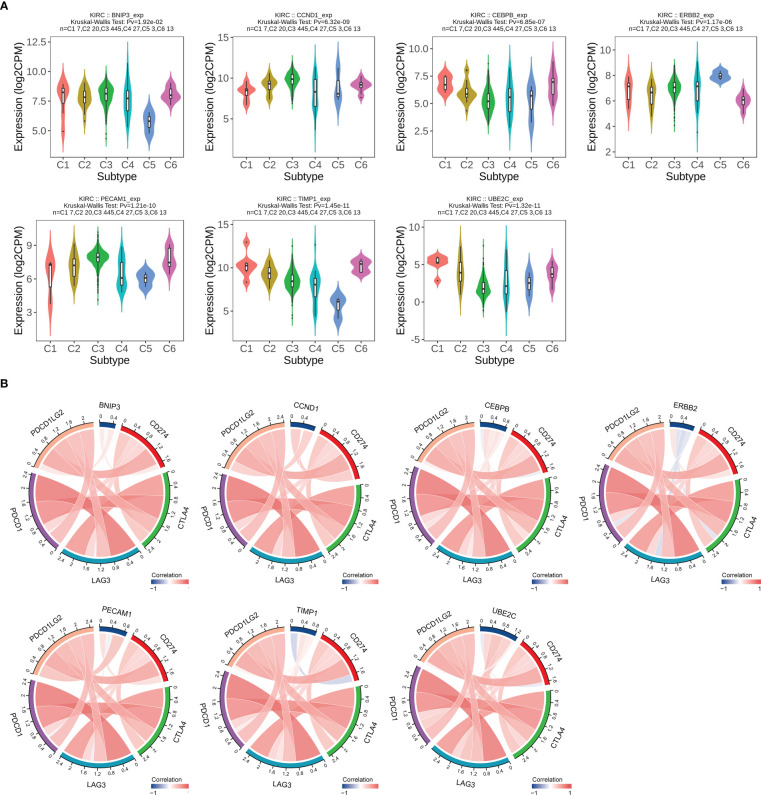
Correlation analysis between ARGs expression levels and immune subtypes and immune checkpoints in ccRCC. **(A)** ARGs expression in different immune subtypes in ccRCC. C1, wound healing; C2, IFN-g dominant; C3, inflammatory; C4, lymphocyte depleted; C5, immunologically quiet; C6, TGF-β dominant. **(B)** Relationship between ARGs expression and main immune checkpoint genes. The red color indicates a positive association, while the blue color represents a negative association.

In addition, we evaluated the relationship between expression of ARGs and immune subtype in ccRCC, and the result displayed high heterogeneity. Immune subtype consisted of six types, including C1 (wound healing), C2 (IFN-gamma dominant), C3 (inflammatory), C4 (lymphocyte depleted), C5 (immunologically quiet), C6 (TGF-β dominant) (25). BNIP3 and UBE2C were highest expressed in C1, CCND1 and PECAM1 were highest expressed in C3, CEBPB and TIMP1 were highest in C6, and ERBB2 was highest expressed in C5 (**Figure 9A**). We also evaluated the relationship between ARGs and significant immune checkpoint genes including CD274 (PD-L1), CTLA4, PDCD1LG2 (PD-L2), LAG3 and PDCD1 (PD-1) in ccRCC. As displayed in **Figure 9B**, the expression levels of CEBPB, TIMP1 and UBE2C were positively correlated with most immune checkpoint genes except for CD274, indicating that they could be potential targets for immunotherapy.

### Correlation and enrichment analysis of ARGs

**Figure 10:**
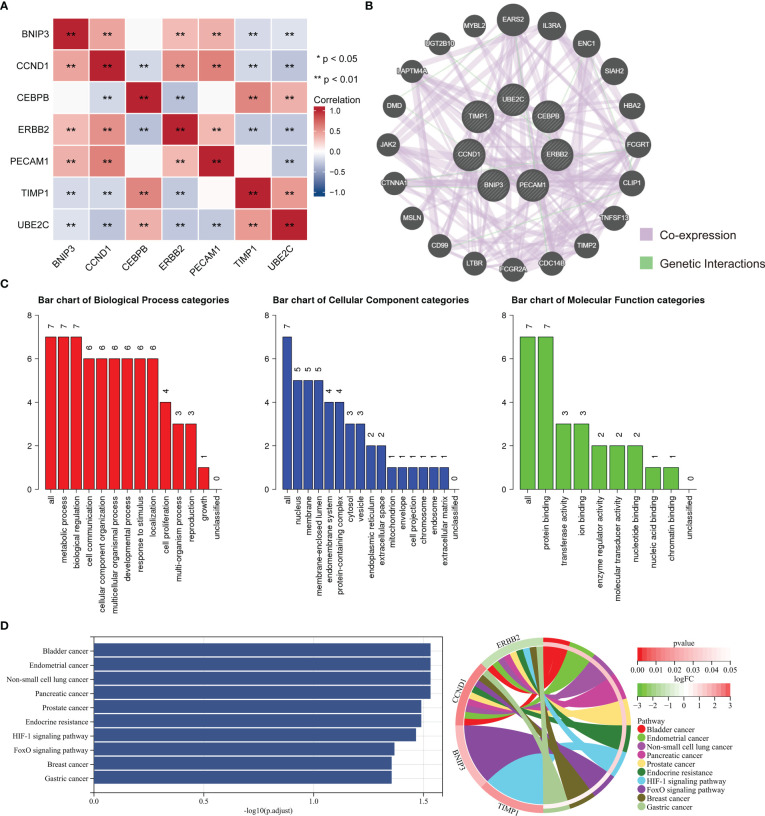
Enrichment analysis of ARGs and related partners. **(A)** The correlation analysis between the ARGs. Blue color represents a negative correlation and red color represents a positive correlation. **(B)** Protein–protein interaction network of the ARGs and relevant genes by GeneMANIA tool. **(C)** GO analysis of related biological processes, cellular components, and molecular functions of ARGs. **(D)** KEGG enrichment analysis of ARGs.

The correlation analysis of ARGs was conducted in ccRCC, and result was displayed in **Figure 10A**. GeneMANIA is an online site which have abundant genetic information and can analyze the correlation among several genes (28). Then, the GeneMANIA website was utilized to explore the several potential interaction partners of ARGs. As showed in **Figure 10B**, the expression of ARGs were closely correlated with EARS2, IL3RA, ENC1 and so on. We next employed GO and KEGG analysis to investigate the biological process, cellular component, molecular function and related signaling pathways of seven ARGs. GO analysis indicated that biological regulation and metabolic process were included in biological process, and protein binding was involved in molecular function (**Figure 10C**). The result of KEGG analysis demonstrated that ARGs were involved in occurrence and development of multiple types of tumors, endocrine resistance, and HIF-1 signaling pathway (**Figure 10D**).

Then, the single cell dataset KIRC_GSE121636 in the TISCH database was employed to explore the expression of ARGs in TME. In the GSE121636 dataset, CEBPB, PECAM1 and TIMP1 were mainly expressed in monocyte, while ERBB2 was almost not detected in immune cell (**Figure S5**).

## Discussion

ccRCC was considered as the most common pathological subtype of urologic malignances (29). Several approaches of treating RCC have been employed in clinical work including surgery, radiofrequency ablation, immunotherapy, chemotherapy, and targeted therapy. After radical or partial nephrectomy, plenty of ccRCC patients still develop distant metastases, contributing to higher mortality (30). No enough prognostic indicator or signature has been verified for the clinical outcome of ccRCC patients. Although doctors have made a remarkable progress in treating ccRCC, the endpoint of advanced and metastatic patients is still not ideal (31). The 3-year survival rate of patients with lymph node metastasis is 20-30% after surgery. Targeted therapy including sunitinib has been utilized to treat advanced RCC patients, while large number of patients could acquire drug resistance within 6-15 months, and the OS remains unsatisfactory (32–34). Therefore, it is necessary to explore novel biomarker as the prognostic indicator or therapy target of ccRCC.

Anoikis is a significant defense mechanism, restraining the readhesion of shedding cells to an inappropriate matrix and inhibiting their aberrant growth (35). Previous studies have illustrated that the occurrence of anoikis relied on extrinsic pathway and intrinsic pathway (36). Anoikis plays a significant role in tumor metastasis and is closely correlated with tumor development and therapy failure (37). Malignance cells deprive from primary tissue and adapt to abnormal growth elsewhere, contributing to tumor migration. Two main strategies have been demonstrated to achieve anoikis resistance in tumor, including the regulation of the cell adhesion molecules expression and employment of oxidative stress (38). Nevertheless, the accurate mechanisms of anoikis resistance in ccRCC are still need for further study. Past evidence has indicated that anoikis-resistant ccRCC cells displayed insensitiveness to detachment-induced apoptosis and uncontrolled proliferation. Meanwhile, the expression of Tyrosine receptor kinase B (TrkB) in them was higher than parental ccRCC cells (39). Quinazoline-based drugs were described to induce anoikis in RCC through activating focal adhesion survival signaling pathway, which could be a novel therapy method of RCC (40). Moreover, an extracellular matrix deprivation system (EDS) was developed to reverse anoikis resistance of RCC cells *via* downregulating FAK phosphorylation (15).

In this study, we constructed and verified the APM containing seven ARGs, namely BNIP3, CCND1, CEBPB, ERBB2, PECAM1, TIMP1, and UBE2C. An increasing number of studies have demonstrated that these genes were strongly correlated with occurrence and pathogenesis of cancer. As a mitophagy receptor, upregulation of BNIP3 was associated with poorer melanoma patient’s endpoint, and BNIP3 knockout in melanoma cells inhibited tumor growth *in vivo* (41). CCND1, a positive cell cycle regulator, was frequently deregulated in tumor and was an indicator of cancer phenotype and disease development (42). CEBPB was found to be involved in aerobic glycolysis and promote growth of breast cancer (43). On the other hand, overexpression of MIR503 host gene regulated invasion and proliferation of cervical cancer cells and accelerated cell apoptosis by miR‐191/CEBPB axis (44). ERBB2 was replicated through elevated gene transcription in various of cancer cells, and tumor growth depended on ERBB2 amplification heavily (45). A series of studies identified that PECAM1 was linked to development of several malignances and had the potential to be a diagnostic and prognostic biomarker in gastric cancer patients (46, 47). The knockdown of TIMP1 expression inhibited metastasis, and proliferation but prompted apoptosis of colon cancer through activating FAK-PI3K/AKT and MAPK pathway (48). And TIMP1 could be an independent prognostic biomarker of OS and disease-free survival for colon cancer patients. As for UBE2C, suppression of UBE2C expression restrained E2-promoted invasion, migration and epithelial mesenchymal transition *in vitro* and *in vivo*, and UBE2C elevation was significantly correlated with higher histologic grade, recurrence, and worse OS in endometrial cancer (49).

The Infiltration of Treg cells was often correlated with worse prognosis in diverse tumors, and decreased Treg cells was found to evoke and promote anti-tumor immune response (50). In our study, the expression levels of BNIP3, CCND1, ERBB2, and PECAM1 were significantly negatively correlated with infiltration level of Treg cells, and CEBPB, TIMP1, and UBE2C were positively associated with infiltration level of Treg cells, suggesting that these genes might affect ccRCC patients’ prognosis through regulating infiltration of Treg cells. To explore the potential mechanisms or functions of the ARGs, functional enrichment analysis was conducted. KEGG analysis demonstrated that the seven ARGs were mainly enriched in several types of malignances, including bladder cancer, endometrial cancer, non-small cell lung cancer, pancreatic cancer, and prostate cancer, indicating the intimate relationship between ARGs and different types of tumors.

In summary, we systematically constructed and assessed the APM of ccRCC based on 7 ARGs, and this model could well predict the OS of ccRCC patients. Moreover, we found that these 7 ARGs were closely correlated with immune cell infiltration and immune checkpoint genes. Our study could provide insights for further exploring the role of ARGs in ccRCC and contribute to the progression of personalized and precise therapy strategies.

## Data availability statement

The datasets presented in this study can be found in online repositories. The names of the repository/repositories and accession number(s) can be found in the article/**Supplementary Material**.

## Author contributions

CS and HS designed the overall study. JZ, ZZ, and ZX revised the paper. YL and ZS drafted the manuscript and performed the data analysis. YB, MF and YD participated in the data collection. All authors contributed to the article and approved the submitted version.

## Glossary

**Table:**

Abbreviation	Full name
APM	Anoikis prognostic model
ARGs	Anoikis-related genes
ATCC	American Type Cultural Collection
AUC	Area under curve
BNIP3	Bcl-2/adenovirus E1B 19kDa interacting protein 3
CCND1	Cyclin D1
ccRCC	Clear cell renal cell carcinoma
CEBPB	CCAAT/enhancer-binding protein beta
CPTAC	Clinical Proteomic Tumor Analysis Consortium
EARS2	Glutamyl-tRNA synthetase 2
EDS	Extracellular matrix deprivation system
ERBB2	Epidermal growth factor receptor 2
FAIM2	Fas apoptotic inhibitory molecule 2
FBS	Fetal bovine serum
GO	Gene Ontology
GTEx	Genotype-Tissue Expression
IHC	Immunohistochemistry
KEGG	Kyoto Encyclopedia of Genes and Genomes
KLF5	Kruppel-like factor 5
K-M	Kaplan-Meier
LASSO	Least absolute shrinkage and selection operator
OS	Overall survival
PECAM1	Platelet endothelial cell adhesion molecule-1
RCC	Renal cell carcinoma
ROC	Receiver operating characteristic
TCGA	The Cancer Genome Atlas
TIMP1	Tissue inhibitor of metalloprotease 1
TME	Tumor microenvironment
TPGs	Tumor prognostic genes
TrKB	Tyrosine receptor kinase B
UBE2C	Ubiquitin-conjugating enzyme E2C
