# Supplementary material for: Establishment and validation of a novel anoikis-related prognostic signature of clear cell renal cell carcinoma
Source: Front Immunol. 2023 Mar 28;14:1171883. doi: 10.3389/fimmu.2023.1171883 (PMC10086373; doi:10.3389/fimmu.2023.1171883)
Supplement: Supplementary file 1 [file DataSheet_1.docx]

**Supplementary Material**

**Supplementary Figures**

**FIGURE S1**


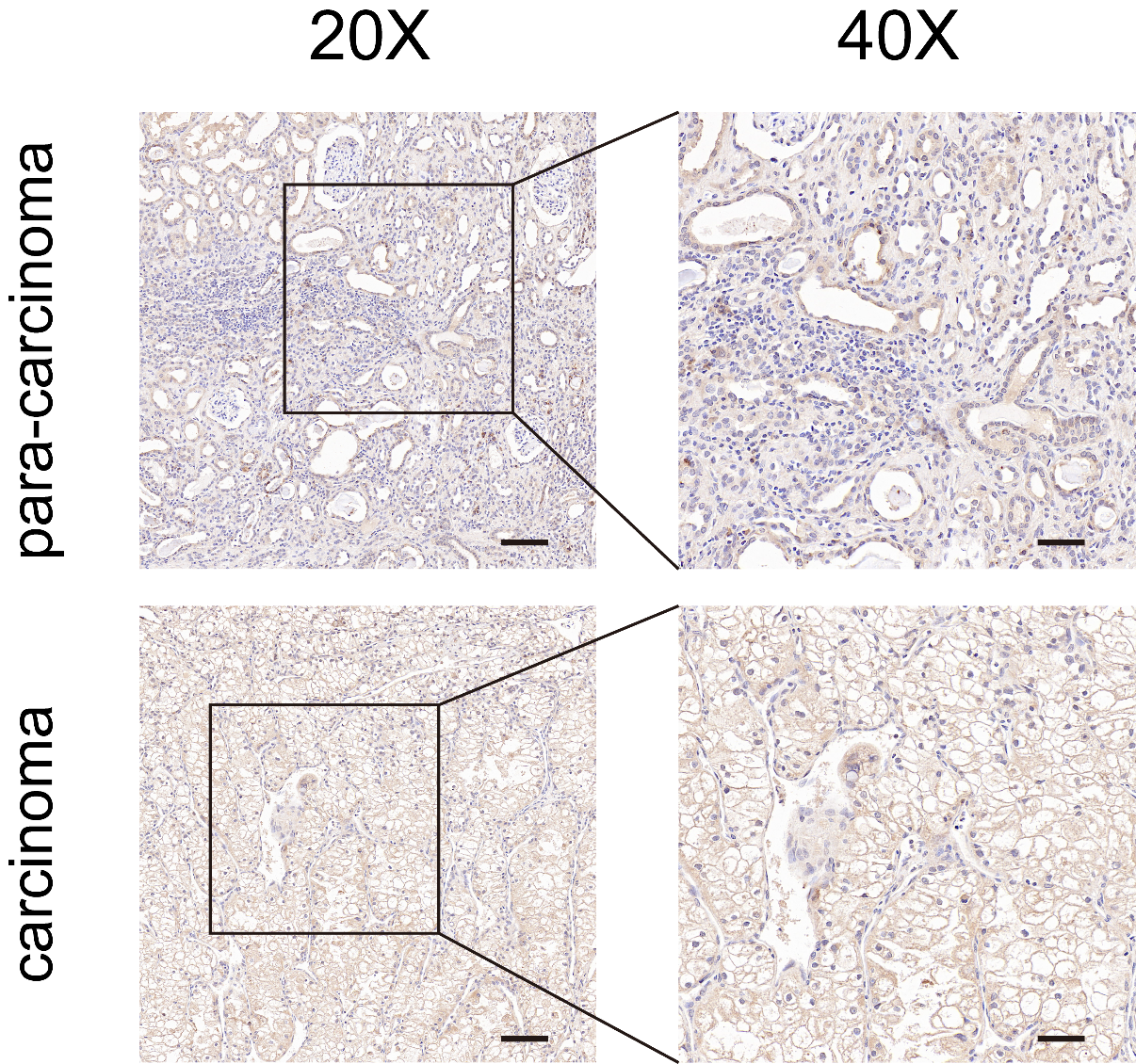


**FIGURE S2**

**
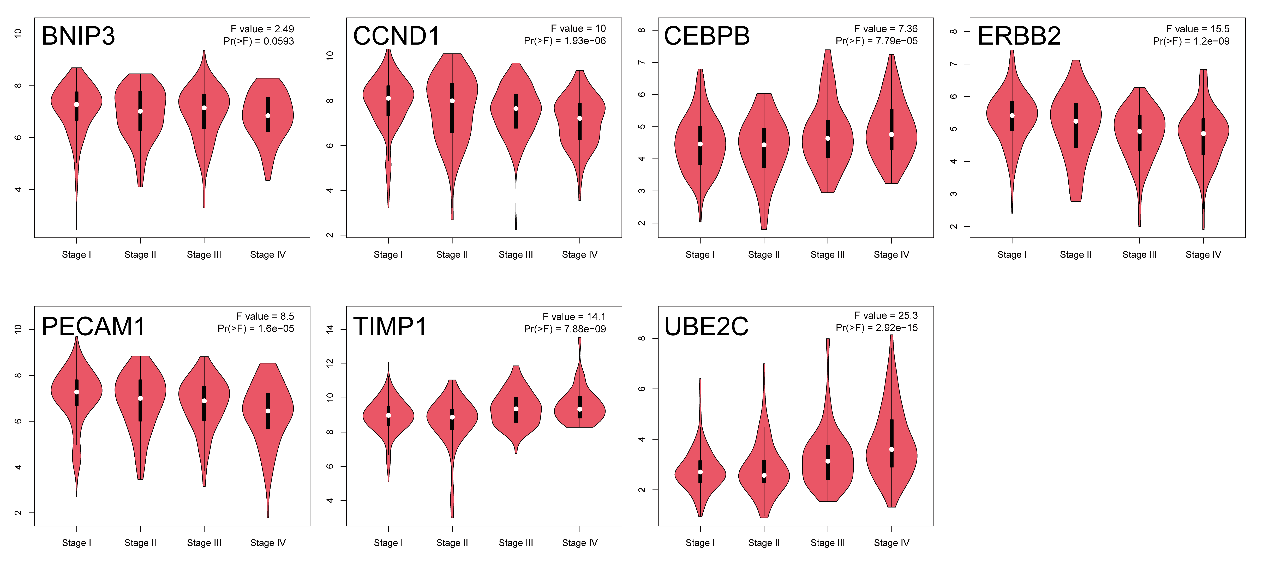
**

**FIGURE S3**

**
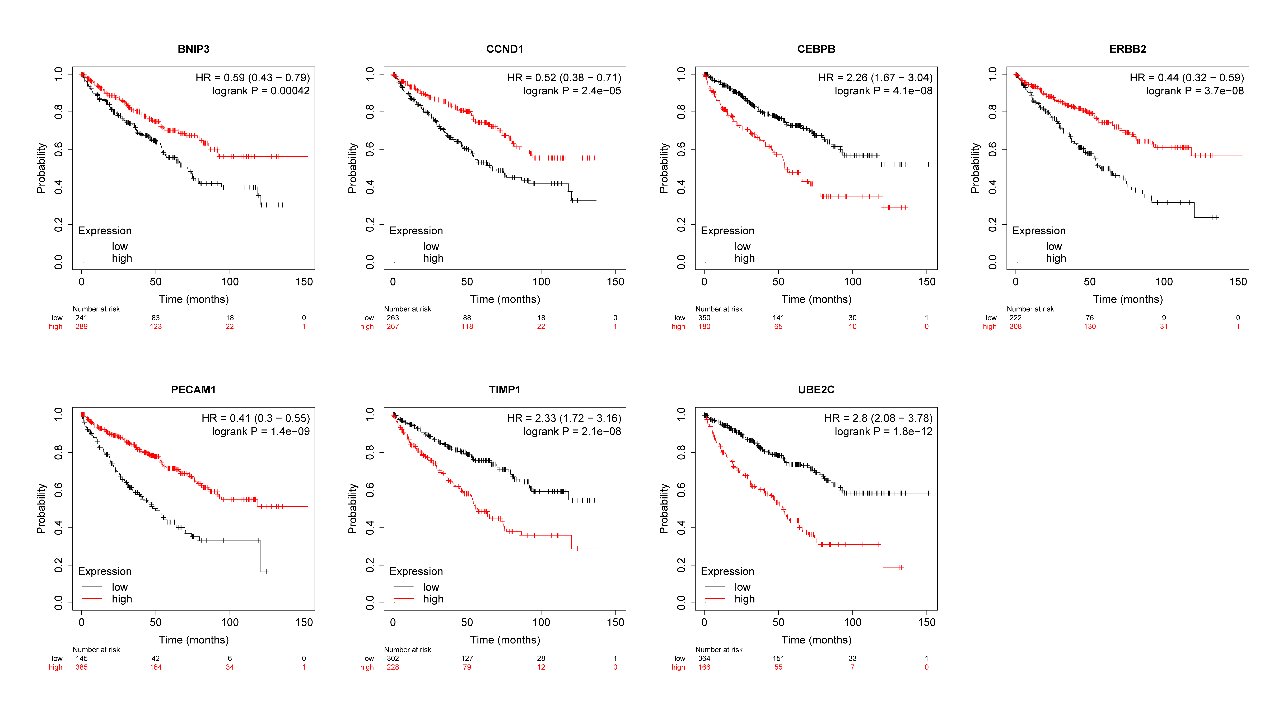
**

**FIGURE S4**

**
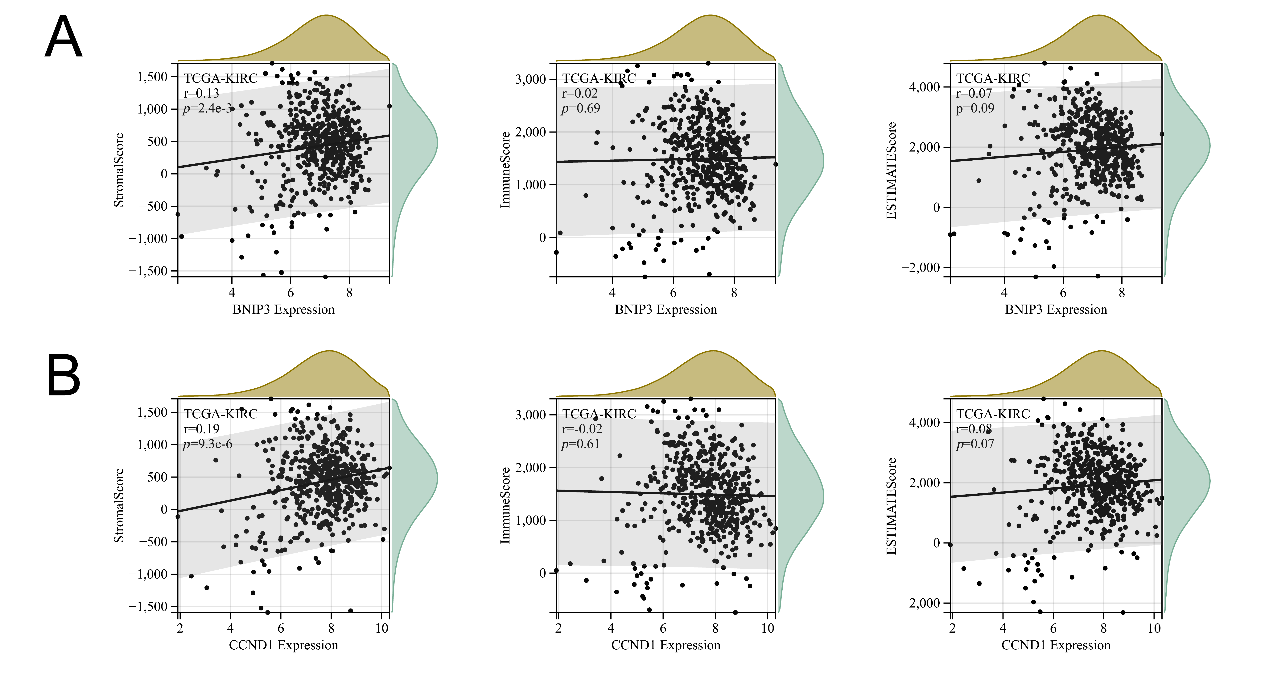
**

**FIGURE S5**

**
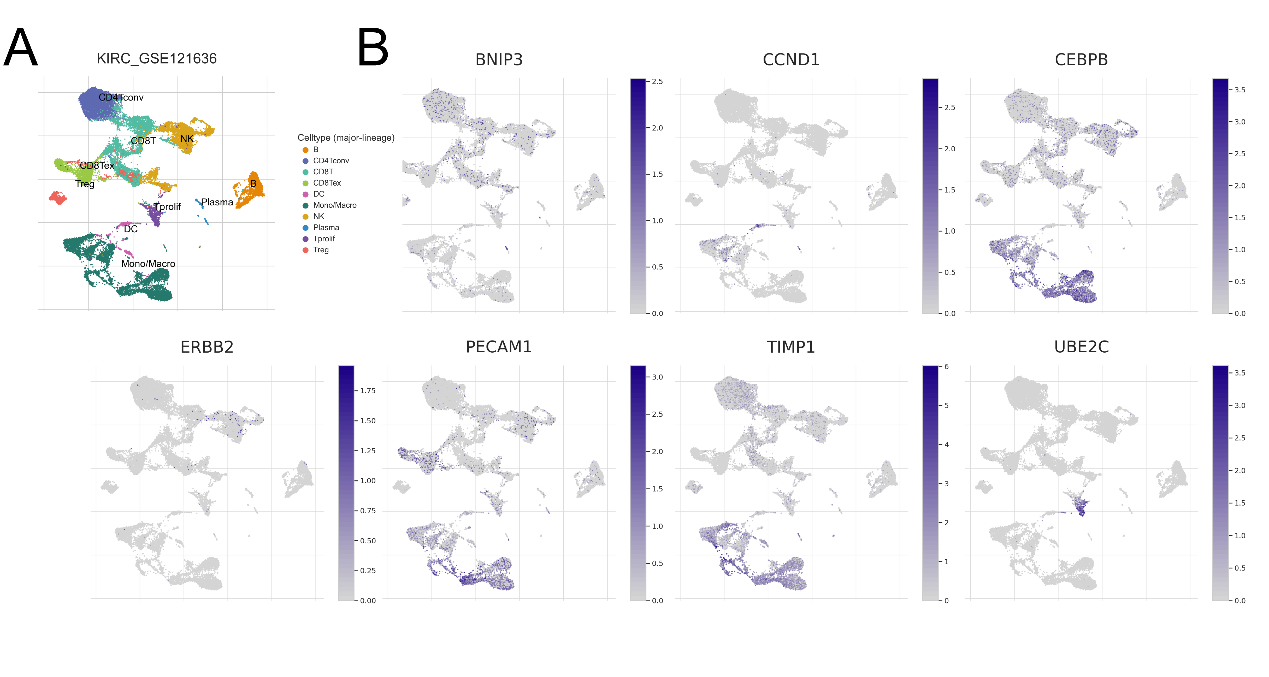
**

**FIGURE S1**

Representative images of immunohistochemistry staining for UBE2C in ccRCC and para-carcinoma tissue. The scale bar in left images was 100um, and in right images was 50um.

**FIGURE S2**

Expression levels of ARGs were assessed and compared by the main pathological stages of ccRCC. The log2(TPM + 1) for log-scale was utilized.

**FIGURE S3**

Relationship between OS and ARGs expression levels in ccRCC. The curves from the K-M plotter demonstrate the independent prognostic value of ARGs. The red lines represent high ARGs expression, while the black lines represent low ARGs expression.

**FIGURE S4**

The correlation analysis between StromalScore, ImmuneScore and ESTIMATEScore and expression levels of (A) BNIP3, (B) CCND1.

**FIGURE S5**

ARGs Expression in ccRCC TME-correlated cells. (A) Annotation of all cell types in GSE121636. (B) The distribution of seven ARGs expression in each cell type.
