# Supplementary material for: Establishment and validation of a novel anoikis-related prognostic signature of clear cell renal cell carcinoma
Source: Front Immunol. 2023 Mar 28;14:1171883. doi: 10.3389/fimmu.2023.1171883 (PMC10086373; doi:10.3389/fimmu.2023.1171883)
Supplement: Supplementary file 2 [file Table_1.docx]

**Table S1:**

Quantitative Real-Time PCR primers used in the study

| **Name** | **Sequences of Primer** |
| --- | --- |
| BNIP3 Forward Primer | CAGGGCTCCTGGGTAGAACT |
| BNIP3 Reverse Primer | CTACTCCGTCCAGACTCATGC |
| CCND1 Forward Primer | CAATGACCCCGCACGATTTC |
| CCND1 Reverse Primer | CATGGAGGGCGGATTGGAA |
| CEBPB Forward Primer | CTTCAGCCCGTACCTGGAG |
| CEBPB Reverse Primer | GGAGAGGAAGTCGTGGTGC |
| ERBB2 Forward Primer | TGCAGGGAAACCTGGAACTC |
| ERBB2 Reverse Primer | ACAGGGGTGGTATTGTTCAGC |
| PECAM1 Forward Primer | ACCGTGACGGAATCCTTCTCT |
| PECAM1 Forward Primer | GCTGGACTCCACTTTGCAC |
| TIMP1 Forward Primer | CTTCTGCAATTCCGACCTCGT |
| TIMP1 Reverse Primer | ACGCTGGTATAAGGTGGTCTG |
| UBE2C Forward Primer | GACCTGAGGTATAAGCTCTCGC |
| UBE2C Reverse Primer | CAGGGCAGACCACTTTTCCTT |
